# Physiotherapy interventions for head and trunk control in children with developmental disabilities: A scoping review protocol

**DOI:** 10.12688/f1000research.123955.1

**Published:** 2022-09-21

**Authors:** Shristi Shakya, Shradha S. Parsekar, Selvam Ramachandran, Shamantha Madapura S., Harikishan Balakrishna Shetty, Dana Anaby, Sivakumar Gopalakrishna, V. S. Venkatesan, Bhamini Krishna Rao

**Affiliations:** 1 Department of Physiotherapy, Manipal College of Health Professions, Manipal, Karnataka, 576104, India; 2 Department of Community Medicine, Kasturba Medical College, Manipal, Karnataka, 576104, India; 3 Department of Biomedical Engineering, Manipal Institute of Technology, Manipal Academy of Higher Education, Manipal, Karnataka, India; 4 School of Physical and Occupational Therapy, McGill University, Montreal, Quebec, Canada; 5 Department of Physiology, Kasturba Medical College, Manipal, Karnataka, 576104, India

**Keywords:** Head control, physiotherapy, postural control, technology-based intervention, trunk control

## Abstract

**Background: **Head and trunk control is prerequisite skill that maximizes engagement and participation in one’s environment by integrating vision, oromotor skill, arm control and respiration. Various physiotherapy and technology-based interventions have been utilized to facilitate head and trunk control in children with developmental disabilities. This scoping review is planned to map and summarize existing studies from the scientific literature on physiotherapy and technology-based interventions for head and trunk control in children with developmental disabilities.

**Methods: **The scoping review will utilize the Joanna Briggs Institute scoping review methodology. The review will cover studies including children and adolescents aged between two years and 17 years 11 months 29 days, with developmental disabilities where in child finds difficulty aligning head and trunk in sitting position, against gravity, for more than a minute. We will include randomized controlled trial (RCT), non-RCT, quasi-experimental trial, and systematic reviews that have employed physiotherapy and technology-based interventions. Database-specific search strategy will be used to search records in Medline (PubMed and Web of Science), Embase, Scopus, CINAHL, PEDro, and Cochrane Library. Additionally, various grey literatures and clinical-trial registries will be searched. Two reviewers, independently, will screen and extract the data. Tables and visual representations will be utilized to present the extracted data.

**Registration details: **The protocol has been registered in Open Science Framework, DOI: 10.17605/OSF.IO/B3RSU (22 ^nd^ August 2022)

## Introduction

Developmental disability can be defined as, “as a severe and chronic disability that manifests before age 22, is attributable to a mental and/or physical impairment, results in substantial functional limitations in three or more major life activities and reflects a need for a combination and sequence of special, individualized services that are of extended duration or lifelong”. ^1^ In 2016, 52.9 million children under the age of five had developmental disability. ^2^ Developmental disability encompasses wide range of conditions such as, Global Developmental Delay (GDD) accounting for 5-15% of prevalence worldwide, ^3^ Cerebral Palsy (CP) affecting 1.5 to 4 out of every 1,000 live births across the world, ^4^ Down Syndrome (DS) accounting 10 to 11 out of every 10,000 live births worldwide, ^5^ and Spinal Muscular Atrophy (SMA) with a global incidence of 1 in 12,000 live birth. ^6^

There are many symptoms of children with developmental disabilities, among them the one that requires immediate attention is child facing difficulty to align head and trunk in a sitting position, against gravity. Head control is the primordial motor skill essential for the development and acquisition of higher-level motor abilities besides their contributory role of integrating vision, regulating breathing patterns, and deglutition. These activities are essential for a child's independence in everyday life, and eventually, quality of life (QOL). ^7^^,^^8^ Trunk control, a component of the postural control framework, involves trunk stability and mobility. During the first year of life, the development of trunk control prepares the child to establish anti-gravity motor control to facilitate independent environmental participation, which is commonly impaired in children with developmental disabilities. A stable trunk aids in orienting the child to his/her environment, allowing the child's social, cognitive, and communicative abilities to develop. ^9^

Given the fact that children with developmental disabilities require regular treatment sessions, therapists may find it challenging to engage the children throughout the rehabilitation program. Therefore, there is a need to develop unique ways and tools to encourage children perform exercises that are both affordable and acceptable. Children with disabilities often gets tired or bored performing monotonous exercises hence, (advanced) technology-based interventions may help build an interactive and child-engaging rehabilitation approaches ^7^^,^^10^^,^^11^ and/or may complement existing strategies. Hence, there is a need to incorporate enjoyable and interactive mode of rehabilitation for the children with developmental disabilities, to involve the children in the rehabilitation session.

With this concept, CanChild developed the “F-words in Childhood Disability” concept, consisting of “Function,” “Family,” “Fitness,” “Fun,” “Friends,” and “Future,” and is a conceptualization and modification of the World Health Organization’s (WHO) (2001) the International Classification of Functioning, Disability, and Health (ICF) framework, ^12^ with the goal of incorporating “fun element” in exercises to make the sessions enjoyable and interactive for the child. ^12^^,^^13^ There are many rehabilitation interventions utilized for improving head and trunk control in children with developmental disabilities including the application of multiple physiotherapy (active and passive) and technology-based interventions (manual and digital), which can augment these outcomes by facilitating enhanced interactions and engagement during therapy sessions. The data file 1 in the Extended data highlight some of the physiotherapy and technology-based interventions. ^13^^,^^14^

In literature, most studies focus on standing and balancing abilities of children with developmental disabilities. However, it is impossible to attain stable balance and posture without addressing the core component involved in such abilities, namely, head and trunk control. Studies suggest that newer devices can be utilized to improve child’s motor control, and children find this kind of practice motivating and fun. ^7^^,^^10^^,^^11^ Studies have also utilized many treatment approaches listed under data file 1. ^14^^,^^15^^,^^17^^,^^18^ Most of the study interventions have predominantly focused on impairments and activity limitations in reporting the facilitation of head and trunk control.

Therefore, we intend to utilize physiotherapy and technology-based intervention for developing head and trunk control in children with developmental disabilities, which carries element of fun, variability, interactivity, engagement, motivation, and sense of accomplishment to the children during their therapy session. Moreover, head and trunk control can be considered as a foundation to integrate vision, oromotor skill, arm control, and respiration, ultimately aiding in improving QOL which is broadly described in the “United Nations Sustainable Developmental Goals 3 – Good Health and Well-being”, ^19^ as well as, the use of available interventions to reduce the burden of persons with disability may help in improving the economic and social burden of the country as mentioned in the “United Nations Sustainable Developmental Goals 10 – Reduced Inequalities”. ^20^

A preliminary literature search on the topic showed that most interventions were limited to medical (medicines, surgery, bone-and-tendon lengthening procedures) or re/habilitation management, focusing grossly on improvement of function and strength in extremities, without paying much attention to underlying measures of postural control. ^21^The components of postural control have been identified as construct in relation to the outcome measures, such as, static, and dynamic stability, functional stability limit, cognitive influences, verticality, and reactive postural control. Only few technology-based interventions such as Virtual Reality (VR), ^21^ Interactive Computer Play (ICP), ^10^ Robotics ^22^ have been used in re/habilitation practice. However, most of the interventions were targeted to gross motor functions rather than focusing on the construct. ^23^

Furthermore, a review aimed at identifying and evaluating physical therapy studies in CP children found that majority of studies intended to reduce “impairments and activity limitations”, with little attention directed to “participation and environmental factors”. ^16^ Another review aimed to understand and describe literature addressing effect of altered postural and segmental trunk control on sitting among children with CP. However, it did not focus on strategies to improve postural and segmental trunk control, and interventional studies were excluded. ^24^ No scoping review exists that summarizes studies assessing physiotherapy and technology-based interventions for postural control measuring core element of gross motor skill acquisition. Most of the reviews were restricted to geographical locations and selective interventions, and none of the reviews focused on the core component of postural and gross motor control i.e., head and trunk control. Therefore, this scoping review is planned to address these gaps by answering following questions.

### Review questions

1. What physiotherapy and technology-based interventions are available that assessed head and trunk control and gross motor functions in children with developmental disabilities?
2. What studies are available that measured effectiveness of the physiotherapy and technology-based interventions on head and trunk control and gross motor functions in children with developmental disabilities?

The first question will map available physiotherapy, which also includes technology-based interventions that assessed head and trunk control in children with developmental disabilities. Through second question, we want to summarize available evidence and gaps in the studies assessing intervention and selected outcomes, which may help research community to either design robust interventional studies (if gap exists) or plan a systematic review (based on availability of evidence). This may also help funders in prioritizing research topics while calling for research proposals.

## Protocol

### Method and analysis

The proposed scoping review will be conducted in accordance with the Joanna Briggs Institute methodology for scoping reviews. ^25^ The protocol has been registered in Open Science Framework, DOI: 10.17605/OSF.IO/B3RSU. ^26^ In the case that the proposed protocol is amended, we will detail the change and provide justifications in the final review.

*Eligibility criteria*

Participants: Studies conducted among children between two years to 17 years 11 months and 29 days, who have developmental or neuromotor impairment, with difficulty to align head and trunk in sitting position, against gravity will be included. This difficulty in head and trunk control is frequently seen in conditions such as developmental delay, CP, DS and SMA. Developmental disabilities may include functional limitations of more than three areas of life activities, such as respiration, deglutition, bimanual activities, and bipedal walking. Studies conducted among children and adolescents who have co-morbidities such as Cortical Visual Impairment (CVI), hearing disability, intellectual disability, terminal illness receiving hospice care, and having a history of seizure disorders will be excluded as some of the physiotherapy interventions may be contraindicated among these children and these children may need medical management. Furthermore, this review will exclude population having acquired conditions affecting head and trunk control.

Concept: We will include studies that assessed effectiveness of physiotherapy including technology-based interventions, which are listed, but not restricted to, the ones mentioned in data file 1 in the Extended data. However, the focus of the interventions should be on head and trunk control. The physiotherapy interventions could be varied and classified as “active interventions”, which includes usage of muscles or joints voluntarily by the participants on command, and “passive interventions”, which includes tools/devices used to passively provide the desired response. Pharmaceutical, complementary, and alternative medicines such as yoga, massage, and acupuncture will be excluded. There is no restriction on the intensity, duration, and provider of the intervention.

The outcomes of our scoping review include head and trunk control, gross motor function and activities of daily living (ADL). Head control can be defined as the capacity to maintain head alignment with body against gravity. Poor head control can be defined as inability to align head with reference to the body against gravity. ^27^ Similarly, the ability to maintain trunk stable in sitting position for more than a minute and use upper extremity for functional movements is defined as trunk control. Poor trunk control can be defined as inability/difficulty to maintain the trunk stable in sitting position or requiring external support. ^9^ Head and trunk control can be measured either qualitatively or with different tools or outcome measures. This scoping review will not impose any restriction on outcome measures.

Data file 2 in the Extended data illustrates conceptual framework of our research describing different physiotherapy interventions, their possible outcomes, and overall impact on children with developmental disabilities.

Context: This scoping review will include studies conducted in a variety of settings, such as outpatient, inpatient, and community, country/region, urban, semi-urban, or rural settings. The scoping review will not have any geographic restrictions.

Types of sources: This review will include randomized controlled trials, non-randomized trials, quasi-experimental trials, and systematic reviews of interventional studies. The editorial, conference abstracts, letters, opinions, and comments will be excluded; however, if these employ the study designs, we will contact the corresponding authors and request to share the full papers to be included in the scoping review.

*Information sources*

All identified records will be collated and uploaded into Zotero, and duplicates will be removed. Rayyan QCRI (Qatar Computing Research Institute, Doha, Qatar), a web application, will be utilized to import unique citations, which permits screening, and administration of important screening statistics among reviewers. ^28^ Two independent reviewers will be screening titles and abstracts against the review’s inclusion criteria. The full texts of chosen citations will be retrieved, and two independent reviewers will assess them against the eligibility criteria in detail. Reasons for excluding full-text articles that do not meet the inclusion criteria will be recorded and reported. At each stage of the selection process, any disagreements between the reviewers will be addressed by discussing with a third reviewer. PRISMA 2020 flow diagram will be utilized to present the screening results in the final scoping review. ^29^

*Search strategy*

A preliminary search of Medline (PubMed) and EMBASE was conducted on 18/06/2022 to identify appropriate keywords. A PubMed search strategy was constructed using identified keywords of relevant publications, data file 3. Furthermore, based on the finalized search strategy, the following databases will be searched: Medline (PubMed and Web of Science), Embase, the Cochrane Library, Scopus, CINAHL Complete, and PEDro. Additionally, we will search other sources (grey literature search and registries) such as, Australasian Academy of Cerebral Palsy and Developmental Medicine (AusACPDM), American Academy for Cerebral Palsy and Developmental Medicine (AACPDM), The WHO, CanChild Centre for Childhood Disability Research, ProQuest, MedNAR, OpenGrey, and clinicaltrial.gov. Furthermore, citation tracking through back referencing and forward citations of the included studies will be carried out. The search strategy will be modified according to the databases with the intention to carry out a comprehensive and up-to-date search. Articles published in English from the inception of databases to the date of the search will be included. Studies will be included irrespective of their publication status, irrespective of peer-reviewed or not, such as journal articles, reports, thesis, and dissertations are eligible for inclusion.

*Data extraction*

Data coding tool will be developed and used to code data from the papers included in the scoping review. Specific details regarding “participants”, “concept”, “context”, “study methods” and “important findings” pertinent to the review questions will be included in the retrieved data. A data coding form will be designed and piloted based on the variables enlisted in Table 1. The ICF framework taxonomy will be utilized to determine the variables to be extracted from included studies. Additional or missing data (if any) from included studies will be obtained by contacting the authors of the articles. If the authors do not respond within two weeks of communication, the studies will be included, and appropriate limitations will be indicated. While reporting the interventions focused on head and trunk control in children with developmental disabilities, Consensus on Exercise Reporting Template (CERT) ^30^ and Template for Intervention Description and Replication (TIDieR) ^31^ will be used.

**Table 1.** Preliminary data coding variables.

S. No.	Details	Information
1.	Bibliographical details	Author, year of publication, publisher details
2.	Study details	Objectives of the study, methodology of the study (design, type of analysis)
3.	Geographical location	Country, Region
4.	Population characteristics	Sample size, age, sex, socioeconomic status
5.	Intervention details	Information of the intervention such as; to whom it was provided, main treatment approaches, structure of the intervention like: length/duration of session, sessions/week; who provided, where provided/settings; experience of the participants during the session (pain, boredom, enjoyment, interactivity)
6.	Outcome	Head control, Trunk control, Gross motor function, Activities of daily living

*Data analysis and presentation*

We will summarize objective 1 by preparing a list of interventions that were used for head and trunk control among children with developmental disabilities. We will summarize these interventions as per CERT and TIDieR. To achieve objective 2, we will map studies that assessed the effectiveness of intervention(s) on outcomes of our interest among children with developmental disabilities. We will group these studies based on study types e.g., RCTs, non-RCTs, and systematic reviews. Similarly, ongoing studies will also be documented.

Qualitative and quantitative findings of the studies will be tabulated. Visual representation (illustrations) will be utilized to highlight demographic details using frequency/percentage distribution of the included studies, details of intervention, type of study design, and country or location. We have preliminary mapped the evidence on physiotherapy and technology-based intervention for head and trunk control of children with developmental disabilities from one of the probably included studies using an example table ( Table 2).

**Table 2.** Mapping of available evidence on rehabilitation interventions for head and trunk control.

Author/Year	Study design and study location	Participant details	Intervention details & Comparison	Outcome details (measurement time points)
Brown, Thompson, & Brizzolara, 2018	**Study design**: Quasi experimental design without comparison group (feasibility study) **Study location:** Texas, USA	**Condition**: Poor head control and a neurologic diagnosis - GMFCS level V and had global delays: Twelve children had CP, one child had FOXG1 syndrome and one child Lennox-Gastaut syndrome **Age**: 3-11 years (mean age 7 years) **Sex**: 6 males, 8 females **Number**: 14 (2 lost to follow up, 2 headpod intolerance and 1 died by 3 months)	**Intervention**: Use of Headpod device ‐ *Session*: 3 times/day for 15 minutes (total of 45 minutes per day) ‐ *Duration*: 6 months ‐ *Provider*: Investigators trained parents to apply the headpods **Comparison**: No comparator group	**Primary outcome**: 1) Head control i. Active time - the child held the head upright against gravity to participate in a motivating activity during a 5-minute video capture (3 & 6 months) ii. Head bobs were counted using the same video captures (3 & 6 months) **Secondary outcomes**: 2) Adherence as determined by daily recording of device use on a monthly log sheet (3 & 6 months) 3) Perceived improvement via a 3-question, 15-point global rating of change (GROC) scale survey with an open-ended comment section (6 months)

Risk of bias assessment, meta-analysis or Grading of Recommendations, Assessment, Development and Evaluations, is out of the scope of our scoping review.

*Patient and public involvement*

We had an interaction with stakeholders (a parent and health professional), to get their feedback on defining the research topics. One of the stakeholders is a parent of a child with developmental disabilities, receiving regular therapy for the acquisition of head and trunk control. Another stakeholder is a pediatric physiotherapist who reviewed our protocol.

*Ethics and dissemination*

We will conduct a scoping review of existing studies and will not recruit participants directly; thus, ethical approval will not be required. We intend to present the results of the full scoping review at an international conference and submit the paper for publication in a peer-reviewed journal.

### Strengths and limitations

• This scoping review will employ a comprehensive search in major databases and other sources (including grey literature search) to identify relevant studies.
• Stakeholders (parent of child with developmental disability and health professional) were consulted to get feedback on this protocol.
• Due to resource limitations, this review will be confined to publications in the English language.

### Study status

We are currently identifying the keywords for search strategy and anticipate running the search in various databases at the end of September 2022.

## Conclusion

This scoping review will emphasize the current research and knowledge gaps in physiotherapy and technology-based interventions for head and trunk control in children with developmental disabilities. Therefore, this information may be used by the scientific community to conduct interventional research or carry out a systematic review. Additionally, this can help funders prioritise research topics when they are seeking research proposal submissions.

## Data availability

### Underlying data

No data are associated with this article.

### Extended data

OSF: Physiotherapy interventions for head and trunk control in children with developmental disabilities - A scoping review protocol. https://doi.org/10.17605/OSF.IO/B3RSU. ^26^

This project contains the following extended data:

- Data file 1 (Supplementary Table 1 listing the rehabilitation interventions used in physiotherapy for children with developmental disabilities)
- Data file 2 (Appendix 1 is the figure illustrating the Conceptual Framework mapping the physiotherapy and technology-based interventions in children with Developmental Disabilities)
- Data file 3 (Appendix 2 is the preliminary PubMed search strategy carried out on 18/06/2022)

## Reporting guidelines

OSF: PRISMA-P checklist for “Physiotherapy interventions for head and trunk control in children with developmental disabilities - A scoping review protocol”. https://doi.org/10.17605/OSF.IO/B3RSU. ^26^

Data are available under the terms of the Creative Commons Attribution 4.0 International license (CC-BY 4.0)
