# Supplementary material for: Physiotherapy interventions for head and trunk control in children with developmental disabilities: A scoping review protocol
Source: F1000Res. 2023 Mar 17;11:1074. Originally published 2022 Sep 21. [Version 2] doi: 10.12688/f1000research.123955.2 (PMC9975404; doi:10.12688/f1000research.123955.2)
Supplement: Peer review history

# Peer review: Reviewer response for version 2

**DOI:** 10.5256/f1000research.145525.r166784

**Published:** 2023-03-22

**Authors:** Jodi Thomas

**Affiliations:** 1 School of Physical Therapy, Texas Woman's University, Dallas, TX, USA

No new comments to add.

Is the study design appropriate for the research question?

Yes

Is the rationale for, and objectives of, the study clearly described?

Yes

Are sufficient details of the methods provided to allow replication by others?

Yes

Are the datasets clearly presented in a useable and accessible format?

Yes

Reviewer Expertise:

Pediatric Physical Therapy-development and validation of a scale to assess head control

I confirm that I have read this submission and believe that I have an appropriate level of expertise to confirm that it is of an acceptable scientific standard.

# Peer review: Reviewer response for version 1

**DOI:** 10.5256/f1000research.136117.r162768

**Published:** 2023-02-28

**Authors:** Emily C. Marcinowski

**Affiliations:** 1 School of Kinesiology, Louisiana State University, Baton Rouge, LA, USA

Overall, I found this review protocol to be organized well and clear with its goals. I’ve include a few comments below that I believe will improve it further:

 Methods:

 (Table 1/3. Geographical Information): I would consider including information about a community’s resources for providing intervention to these populations (e.g., health care structure, access to Early Intervention). By providing this, readers can see the standard of care of a study’s region and potentially describe accessibility and efficacy of a technology-based intervention.

 (Protocol / Eligibility criteria): “Furthermore, this review will exclude population having acquired conditions affecting head and trunk control.” Can you give some examples of what these would include?

 (Protocol / Patient and public involvement): Please add a brief description of what the stakeholders provided to the review. Information on scope of the review?  Terminology?  Labor?  Priorities?

 Conclusion:

 You primarily discuss this review is to fill in the gaps to improve interventional research and prioritize funding goals. I think it makes sense to describe more fully how these findings lend themselves to quality, evidence-based practices for technology-based interventions. Technology is unique in that it can improve or limit accessibility to the community, depending on its cost, necessary equipment, and location/community resources available. By widening your discussion points to include this point, I think it would widen your audience and make a better point for researchers and funders to consider what is necessary for technology-based interventions.

Is the study design appropriate for the research question?

Yes

Is the rationale for, and objectives of, the study clearly described?

Yes

Are sufficient details of the methods provided to allow replication by others?

Yes

Are the datasets clearly presented in a useable and accessible format?

Yes

Reviewer Expertise:

motor development, infancy, active play, motor delays or disorders, cultural variations in dyadic play and development

I confirm that I have read this submission and believe that I have an appropriate level of expertise to confirm that it is of an acceptable scientific standard.

# Peer review: Reviewer response for version 1

**DOI:** 10.5256/f1000research.136117.r161522

**Published:** 2023-02-14

**Authors:** Jodi Thomas

**Affiliations:** 1 School of Physical Therapy, Texas Woman's University, Dallas, TX, USA

The article outlines the planned protocol for this scoping review well. The steps that will be taken are defined and clear. This scoping review aims to find studies regarding physiotherapy and technology based interventions that are used in the treatment of head and neck control deficits. The authors mention the need to address ICF components such as activity limitations; I think it would be beneficial for the data extraction to include how the intervention addressed ICF components. One item that is unclear to me is why the age range is set at 2 years, excluding articles that include children younger than 2 years. The rationale for this was not made clear. Overall, this protocol demonstrates sound scientific design and should results in information that is useful to the physiotherapy community as well as many others.

Is the study design appropriate for the research question?

Yes

Is the rationale for, and objectives of, the study clearly described?

Yes

Are sufficient details of the methods provided to allow replication by others?

Yes

Are the datasets clearly presented in a useable and accessible format?

Yes

Reviewer Expertise:

Pediatric Physical Therapy-development and validation of a scale to assess head control

I confirm that I have read this submission and believe that I have an appropriate level of expertise to confirm that it is of an acceptable scientific standard.
